# The effect of a preparation of minerals, vitamins and trace elements on the cardiac gene expression pattern in male diabetic rats

**DOI:** 10.1186/s12933-015-0248-6

**Published:** 2015-06-28

**Authors:** Márta Sárközy, Gergő Szűcs, Márton Pipicz, Ágnes Zvara, Katalin Éder, Veronika Fekete, Csilla Szűcs, Judit Bárkányi, Csaba Csonka, László G. Puskás, Csaba Kónya, Péter Ferdinandy, Tamás Csont

**Affiliations:** Department of Biochemistry, Faculty of Medicine, University of Szeged, Szeged, Hungary; Institute of Genetics, Biological Research Center of the Hungarian Academy of Sciences, Szeged, Hungary; Department of Genetics, Cell- and Immunobiology, Semmelweis University, Budapest, Hungary; Béres Pharmaceuticals Ltd, Budapest, Hungary; Pharmahungary Group, Szeged, Hungary; Department of Pharmacology and Pharmacotherapy, Semmelweis University, Budapest, Hungary

**Keywords:** Diabetes mellitus, Haemoglobin A1c, Insulin resistance, Multivitamin, Heart, DNA microarray, Cardiac hypertrophy, Fibrosis, Oxidative/nitrative stress

## Abstract

**Background:**

Diabetic patients have an increased risk of developing cardiovascular diseases, which are the leading cause of death in developed countries. Although multivitamin products are widely used as dietary supplements, the effects of these products have not been investigated in the diabetic heart yet. Therefore, here we investigated if a preparation of different minerals, vitamins, and trace elements (MVT) affects the cardiac gene expression pattern in experimental diabetes.

**Methods:**

Two-day old male Wistar rats were injected with streptozotocin (i.p. 100 mg/kg) or citrate buffer to induce diabetes. From weeks 4 to 12, rats were fed with a vehicle or a MVT preparation. Fasting blood glucose measurement and oral glucose tolerance test were performed at week 12, and then total RNA was isolated from the myocardium and assayed by rat oligonucleotide microarray for 41012 oligonucleotides.

**Results:**

Significantly elevated fasting blood glucose concentration and impaired glucose tolerance were markedly improved by MVT-treatment in diabetic rats at week 12. Genes with significantly altered expression due to diabetes include functional clusters related to cardiac hypertrophy (e.g. *caspase recruitment domain family, member 9; cytochrome P450, family 26, subfamily B, polypeptide; FXYD domain containing ion transport regulator 3*), stress response (e.g. *metallothionein 1a; metallothionein 2a; interleukin-6 receptor; heme oxygenase (decycling) 1; and glutathione S-transferase, theta 3*)*,* and hormones associated with insulin resistance (e.g. *resistin; FK506 binding protein 5; galanin/GMAP prepropeptide*). Moreover the expression of some other genes with no definite cardiac function was also changed such as e.g. *similar to apolipoprotein L2*; *brain expressed X-linked 1; prostaglandin b2 synthase (brain).* MVT-treatment in diabetic rats showed opposite gene expression changes in the cases of 19 genes associated with diabetic cardiomyopathy. In healthy hearts, MVT-treatment resulted in cardiac gene expression changes mostly related to immune response (e.g. *complement factor B*; *complement component 4a; interferon regulatory factor 7; hepcidin).*

**Conclusions:**

MVT-treatment improved diagnostic markers of diabetes. This is the first demonstration that MVT-treatment significantly alters cardiac gene expression profile in both control and diabetic rats. Our results and further studies exploring the mechanistic role of individual genes may contribute to the prevention or diagnosis of cardiac complications in diabetes.

**Electronic supplementary material:**

The online version of this article (doi:10.1186/s12933-015-0248-6) contains supplementary material, which is available to authorized users.

## Background

In 2014, the global prevalence of diabetes mellitus (DM) was estimated to be 9 % among adults aged over 18 years [1] reaching epidemic rates in the 21st century [2]. The number of patients diagnosed with DM is continuously increasing due to the increasing prevalence of hyperlipidaemia, visceral obesity and physical inactivity worldwide [2–4]. The total number of people suffering from DM is projected to rise to 552 million in 2030 [5].

Regular consumption of complex preparations containing various vitamins, minerals, and trace elements (MVT) is common in developed countries [6] to maintain general health. In the USA, for instance, more than half of the adult population use dietary supplements [7, 8], primarily in the form of multivitamins with or without minerals [9, 10]. In 1998 a study reported that in Germany 18 % of men and 25 % of women were regular consumers of multivitamins among 18–79 years old adults [11]. Moreover, MVT preparations appeared on the market as medical food for patients suffering from metabolic diseases including hyperlipidaemia, metabolic syndrome, and DM. However, the literature is limited and controversial on the potential beneficial effects of these complex preparations containing more than 3 components on disease progression [12–16]. We have recently shown that chronic treatment with a MVT preparation improved well-established diagnostic markers of DM such as fasting blood glucose, HbA1c, glucose tolerance, and serum insulin levels in male diabetic rats [14].

It is well known that diabetic patients have an increased risk of developing cardiovascular diseases including diabetic cardiomyopathy [17]. Cardiovascular complications are estimated to be responsible for more than 50 % of deaths among this population [18]. To explain the development of cardiovascular complications, the effect of DM on the cardiac gene expression pattern was investigated in a few studies [19–22]. In addition, we have previously shown that metabolic syndrome, which is a precursor state of type 2 DM, alters cardiac gene expression pattern in male ZDF rats [23]. However, the effect of MVT preparations on the cardiac gene expression pattern either in healthy or in diabetic condition has not yet been investigated.

Therefore, here we aimed at investigating the effects of a MVT preparation containing 26 different minerals, vitamins and vitamin-like antioxidants, as well as trace elements on the cardiac gene expression pattern in male diabetic rats.

## Materials and methods

### Animals

This investigation conforms to the National Institutes of Health Guide for the Care and Use of Laboratory Animals (NIH Pub. No. 85–23, Revised 1996) and was approved by the Animal Research Ethics Committee of the University of Szeged.

Two-day old neonatal male Wistar rats were used in this study. Lactating females with their litters were separately housed in individually ventilated cages (Sealsafe IVC system, Italy) and were maintained in a temperature-controlled room using 12:12 h light:dark cycles for 4 weeks. After separation from the mother at week 4, males were fed with standard rat chow (Additional file 1) and housed in pairs under the same circumstances as mentioned above until 12 weeks of age.

The injection of neonatal rats with streptozotocin (STZ) is a well characterized and recognized model of diabetes mellitus [24–27]. This model has been particularly characterized in detail in our previous study [14]. STZ is toxic especially to pancreatic β-cells. In contrast to injection of STZ to adult rats in a lower dose (60 mg/kg) that leads to type 1 diabetes with severe hyperglycemia [14, 24–27], administration of STZ to neonatal rats in a higher dose (90–100 mg/kg) leads to acute hyperglycemia within the first few days without resulting in complete loss of insulin production [14, 24–27]. According to literature data, after 5–6 weeks, the surviving rats develop characteristic symptoms of type 2 diabetes mellitus including dyslipidemia, hyperglycemia, impaired glucose tolerance and insulin resistance due to decreased β-cell mass [14, 24–27]. After 10–12 weeks, cardiovascular complications including hypertension and LV hypertrophy with decreased cardiac function are present in neonatal STZ-treated rats [28–34]. Therefore, this model is an appropriate choice to perform anti-diabetic drug screening studies.

### Experimental protocol

Two days old neonatal male Wistar rats were intraperitoneally injected with 100 mg/kg of STZ (*n* = 25) or its vehicle (ice-cold citrate buffer, *n* = 16) to induce experimental DM as described earlier [14].

At week 4, fasting blood glucose (FBG) measurement followed by an oral glucose tolerance test (OGTT) were performed in order to verify the development of impaired glucose tolerance in DM in surviving animals (data not shown). Mortality rates were 0 % and 36 % in citrate buffer-treated and in the STZ-treated group, respectively. Both citrate buffer-treated (*n* = 16) and STZ-treated rats (*n* = 16) were then supplemented per os via gavage (1 mL/kg, 1 % methylcellulose suspension) with a MVT preparation (253.3 mg/kg/day, suspended in methylcellulose, *n* = 8-8) or its vehicle (157 mg/kg/day, suspended in methylcellulose, *n* = 8-8) for 8 weeks as described previously [14]. The MVT preparation administered in the present study is a registered medical food for human use (Diacomplex film-coated tablet, Béres Pharmaceuticals, Budapest, Hungary; for content see Table 1). To conform to the human daily dose of the preparation, the daily rat dose was adjusted according to the ratio of human and rat body surface areas as described previously [14]. Body weight was monitored every week. FBG, haemoglobin A1c level measurement and OGTT were performed at week 12 to assess the effect of MVT-treatment on DM. Serum insulin measurements were performed at week 4, 8 and 12 in order to monitor the insulin secretion of pancreatic beta cells and the effect of MVT-treatment on the severity of pancreatic beta cell damage due to STZ-treatment. At week 12, the rats were anaesthetized using pentobarbital (Euthasol, i.p. 50 mg/kg; Produlab Pharma b.v., Raamsdonksveer, The Netherlands) [35]. Hearts and pancreata were isolated, and then the hearts were perfused according to Langendorff as described earlier [36, 37]. Coronary flow was measured during the ex vivo perfusion protocol [35]. After 10 min perfusion, ventricular tissue was frozen and stored at −80 °C until DNA microarray investigation and gene expression analysis could be performed. Pancreata were removed, trimmed free of adipose tissue, then frozen and stored for pancreatic insulin content determination as described previously [23].

**Table 1:** Ingredients of the MVT preparation

Active ingredients	Amount of ingredient/1 g product	Daily dose^a^
Vitamin A_1_ (Retinol)	329 μg/g (109700 IU/g)	83.3 μg/kg/day (278 IU/kg/day)
Vitamin B_1_ (Thiamin)	2.30 mg/g	0.58 mg/kg/day
Vitamin B_2_ (Riboflavin)	2.63 mg/g	0.67 mg/kg/day
Vitamin B_3_ (Nicotinamide)	11.8 mg/g	2.99 mg/kg/day
Vitamin B_5_ (Pantothenic acid)	3.95 mg/g	1.00 mg/kg/day
Vitamin B_6_ (Pyridoxine)	3.29 mg/g	0.83 mg/kg/day
Vitamin B_12_ (Cyanocobalamin)	3 μg/g	0.76 μg/kg/day
Folic acid	197 μg/g	49.9 μg/kg/day
Biotin	99 μg/g	25.1 μg/kg/day
Vitamin D_3_ (Cholecalciferol)	3 μg/g (120 IU/g)	0.76 μg/kg/day (30.4 IU/kg/day)
Vitamin K_1_ (Phyllokinone)	26 μg/g	6.59 μg/kg/day
Rutoside	3.29 mg/g	0.83 mg/kg/day
Vitamin C	65.8 mg/g	16.7 mg/kg/day
Vitamin E	32.9 mg/g	8.33 mg/kg/day
Lutein	1.97 mg/g	0.50 mg/kg/day
Chrome	39 μg/g	9.88 μg/kg/day
Zinc	9.87 mg/g	2.50 mg/kg/day
Selenium	26 μg/g	6.59 μg/kg/day
Iron	2.63 mg/g	0.67 mg/kg/day
Iodine	66 μg/g	16.7 μg/kg/day
Manganese	0.66 mg/g	0.17 mg/kg/day
Copper	921 μg/g	233 μg/kg/day
Molybdenum	49 μg/g	12.4 μg/kg/day
Magnesium	65.8 mg/g	16.7 mg/kg/day
Calcium	132 mg/g	33.4 mg/kg/day
Phosphorus	102 mg/g	25.8 mg/kg/day

^a^To conform to the human daily dose of the preparation, rat daily dose was adjusted according to the ratio of human and rat body surface areas

### FBG measurements and OGTT

Rats were fasted overnight (12 h) prior to blood glucose level measurement and OGTT (week 12) in order to verify the development of DM and to monitor the effect of MVT-treatment on the progression of DM as described previously [14]. Briefly, blood samples were collected from the saphenous vein and blood glucose levels were measured using Accucheck blood glucose monitoring systems (Roche Diagnostics Corporation, USA, Indianapolis) [14, 23]. After measurement of baseline glucose concentrations, glucose at 1.5 g/kg body weight was administered via oral gavage and plasma glucose levels were measured 30, 60 and 120 minutes later during OGTT [14, 23].

### Haemoglobin A1c measurement

To monitor the effect of the MVT containing preparation on the severity of DM, haemoglobin A1c was measured from whole venous blood with an in vitro test (Bio-Rad in2it System) according to the instructions of the manufacturer [14]. The test is based on single wave length photometry (440 nm) to detect glycated fraction separated from the non-glycated fraction by boronate affinity chromatography [14].

### Measurement of serum and pancreatic insulin levels

To monitor the effect of MVT-treatment on the severity of DM, serum and pancreatic insulin levels were measured by an enzyme immunoassay (Mercodia, Ultrasensitive Rat Insulin ELISA) in duplicates according to the manufacturer’s instructions as described previously [14, 23].

### RNA preparation and DNA microarray analysis

Total RNA was isolated from heart samples with Qiagen miRNeasy Mini Kit according to the manufacturer’s protocol (Qiagen, Hilden, Germany). On-column DNase digestion was carried out with the RNase-Free DNase Set (Qiagen GmbH). RNA concentration was measured by NanoDrop 1000 Spectrophotometer (Thermo Fisher Scientific Inc., Waltham, MA, USA) and RNA integrity was determined by an Agilent 2100 Bioanalyzer System (Agilent Technologies Inc., Santa Clara, CA, USA). Samples with an RNA integrity number (RIN) above 8.0 were used for further analysis. RNA was stored at −80 °C until use.

Total RNA (200 ng) was labelled and amplified using the Low Input Quick Amp Labelling Kit according to the instructions of the manufacturer. Labelled RNA was purified and hybridized to Agilent Whole Rat Genome 4x44K array slides, according to the manufacturer’s protocol. After washing, array scanning and feature extraction was performed with default scenario by Agilent DNA Microarray Scanner and Feature Extraction Software 11.01.

### Messenger RNA (mRNA) expression profiling by qRT-PCR

In order to validate gene expression changes obtained by DNA microarray, qRT-PCR was performed on a RotorGene 3000 instrument (Corbett Research, Sydney, Australia) with gene-specific primers and SybrGreen protocol to monitor gene expression as described earlier [23]. Briefly, 2 μg of total RNA was reverse transcribed using the High-Capacity cDNA Archive Kit (Applied Biosystems Foster City, CA, USA) according to the manufacturer’s instructions in a final volume of 30 μL. After dilution with 30 μL of water, 1 μL of the diluted reaction mix was used as a template in the qRT-PCR with FastStart SYBR Green Master mix (Roche Applied Science, Mannheim, Germany) with the following protocol: 10 min at 95 °C followed by 45 cycles of 95 °C for 15 sec, 60 °C for 25 sec and 72 °C for 25 sec. The fluorescence intensity of SybrGreen dye was detected after each amplification step. Melting temperature analysis was done after each reaction to check the quality of the products. Primers were designed using the online Roche Universal Probe Library Assay Design Center. The quality of the primers was verified by MS analysis provided by Bioneer (Daejeon, Korea). Relative expression ratios were calculated as normalized ratios to rat HPRT and Cyclophyllin genes. A non-template control sample was used for each primer to check primer-dimer formation. The final relative gene expression ratios were calculated as delta-delta Ct values. Fold change refers to 2^-ΔΔCt^ (in the case of up-regulated genes) and –(1/2^-ΔΔCt^) (in the case of down-regulated genes).

### Statistical analysis

Statistical analysis was performed by using Sigmaplot 12.0 for Windows (Systat Software Inc). All values are presented as mean ± SEM. Two-Way ANOVA was used to determine the effect of DM or MVT on body weight, FBG, glucose levels during OGTT, OGTT AUC, HbA1c, serum and pancreatic insulin concentrations, pancreas weight and coronary flow. After ANOVA, all pairwise multiple comparison procedures with *Holm-Šídák post hoc* tests were used as multiple range tests. *P* < 0.05 was accepted as a statistically significant difference.

In the microarray experiments, biological and technical replica tests were carried out to gain raw data for statistical analysis. Altogether 9 individual parallel gene activity comparisons were done between two groups. Both in the microarray and qRT-PCR experiments, a two-sample *t*-test was used and the p value was determined to find significant gene expression changes in 3 separate comparisons. In the microarray experiments, a corrected p value was determined for each gene to control the false discovery rate using the *Benjamini and Hochberg* multiple testing correction protocol. Gene expression ratios with p value of <0.05 and log_2_ ratio of < −0.85 or log_2_ ratio of >0.85 (~1.8 fold change) were considered as repression or overexpression respectively in gene activity.

## Results

### Characterization of experimental DM and the effects of MVT-treatment on the progression of DM

**Fig. 1:**
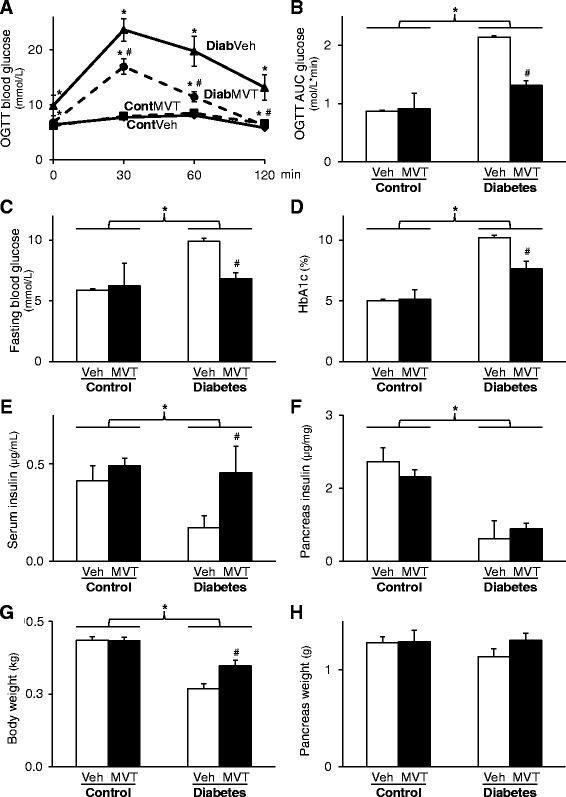
*Characterization of experimental DM and the effects of MVT-treatment on the progression of DM.* **a** Blood glucose levels during oral glucose tolerance test (OGTT) and **b** its area under the curve (AUC). **c** Level of fasting blood glucose and **d** haemoglobin A1c. **e** Serum and **f** pancreas insulin. **g** Body and **h** pancreas weight. Values are expressed as mean ± S.E.M. *p < 0.05 vs. control, #*p* < 0.05 vs. diabetes vehicle, two-way ANOVA, n = 8 in each group. Cont = Control, Diab = Diabetes, Veh = Vehicle, MVT = preparation of minerals, vitamins, and trace elements

The time dependence of development of DM in neonatal STZ-treated rats in both genders has been particularly characterized in detail in our previous study [14]. In the present study, concentrations of several plasma metabolites and body weight were measured in order to verify the development of DM in the STZ-treated rats (Fig. 1). OGTT showed increased glucose levels at every time point following oral glucose load accompanied with increased area under the curve (AUC) values in STZ-treated groups showing impaired glucose tolerance (Fig. 1a and b). MVT-treatment significantly decreased glucose and OGTT AUC values in the STZ-treated groups, proving an anti-diabetic effect of the MVT preparation (Fig. 1a and b). FBG level was significantly higher in STZ-treated groups as compared to the control group showing the development of DM (Fig. 1c). However, FBG level was significantly decreased by MVT-treatment in the STZ-treated diabetic group (Fig. 1c). HbA1c level was significantly increased in STZ-treated groups as compared to controls (Fig. 1d) demonstrating chronic hyperglycaemia and the development of DM. Interestingly, MVT-treatment significantly reduced the HbA1c level in the STZ-treated diabetic group (Fig. 1d). Serum insulin levels were significantly decreased in the STZ-injected vehicle-treated group as compared to control vehicle-treated group both at week 4 (0.05 ± 0.01 vs. 0.16 ± 0.02 μg/mL) and 8 (0.08 ± 0.02 vs. 0.17 ± 0.02 μg/mL) proving deteriorated pancreatic beta cell function in DM. MVT-treatment had no significant effect on serum insulin levels at weeks 4 and 8 in diabetic (0.06 ± 0.01 and 0.12 ± 0.03 μg/mL, respectively) and control animals (0.12 ± 0.02 and 0.25 ± 0.03 μg/mL) when compared to appropriate vehicle-treated controls. At week 12, serum and pancreatic insulin concentration were significantly decreased in STZ-treated diabetic animals, proving pancreatic β-cell damage (Fig. 1e and f). MVT-treatment showed a significant increase in serum insulin concentrations in STZ-treated animals (Fig. 1e). However, MVT-treatment failed to significantly improve pancreatic insulin content in STZ-treated diabetic animals (Fig. 1f). In addition, body weight gain of STZ-treated rats was significantly lower as compared to control rats. However, weight gain was significantly improved by the MVT-treatment in the STZ-treated group (Fig. 1g). Neither DM nor MVT-treatment had a significant effect on pancreas weight (Fig. 1h). Coronary flow was significantly lower in the diabetic vehicle-treated group as compared to the control vehicle-treated group (16.4 ± 2.04 vs. 19.25 ± 0.48 mL/min) showing impaired cardiac function in diabetic hearts. However, MVT-treatment failed to improve coronary flow in the diabetic group (17.0 ± 0.52 mL/min) and had no significant effect in the control group either (19.29 ± 0.94 mL/min).

### Global cardiac gene expression changes

To determine genes in 1) diabetic vehicle-treated, 2) diabetic MVT-treated, 3) control vehicle-treated and 4) control MVT-treated groups, total RNA isolation and then DNA microarray analysis were performed from the hearts of all groups. Among the 41012 rat oligonucleotides surveyed, 16752 oligonucleotides in diabetic vehicle-treated group, 17531 oligonucleotides in diabetic MVT-treated group, 17447 oligonucleotides in control vehicle-treated group and 17184 oligonucleotides in control MVT-treated group showed expression.

### Identification of genes associated with DM

To determine genes associated with DM and diabetic cardiomyopathy, cardiac gene expression induced in the diabetic vehicle-treated group was compared with the control vehicle-treated group (Tables 2 and 3). In diabetic vehicle-treated hearts, 37 genes showed significant up-regulation and 22 genes showed significant down-regulation (Tables 2 and 3).

**Table 2:** Genes significantly down-regulated in diabetes vehicle-treated vs. control vehicle-treated group on DNA microarray

				Diabetes Vehicle			Diabetes MVT		Control MVT	
				vs.			vs.		vs.	
				Control Vehicle			Diabetes Vehicle		Control Vehicle	
Gene function	Gene Name *provided by RGD* ^*a*^	Acc. Number	*Gene Symbol*	Log_2_ (SD)	Corr. *p*	Fold change	Log_2_ (SD)	Fold Change	Log_2_ (SD)	Fold Change
Metabolism	UDP-Gal:betaGlcNAc beta 1,3-galactosyltransferase, polypeptide 2	NM_001109492	*B3galt2*	-1.14 (0.19)	0.003	-2.21	0.80 (0.49)	1.74	-0.16 (0.60)	-1.11
Metabolism	adenylate kinase 4	NM_017135	*Ak4*	-0.99 (0.49)	0.026	-1.99	0.76 (0.40)	1.70	-0.47 (0.70)	-1.38
Stress response	APEX (apurinic/apyrimidinic) endonuclease 2	NM_001079892	*Apex2*	-0.94 (0.56)	0.042	-1.92	0.09 (0.26)	1.06	-1.18 (0.63)	-2.26
Apoptosis/necrosis and inflammation	caspase recruitment domain family, member 9	NM_022303	*Card9*	-2.74 (0.64)	0.003	-6.69	1.82 (0.52)	3.53	-1.20^*b*^ (0.88)	-2.29
Apoptosis/necrosis and inflammation	chemokine (C-X-C motif) ligand 12	NM_001033883	*Cxcl12*	-0.86 (0.38)	0.021	-1.82	0.48 (0.40)	1.40	0.06 (0.30)	1.04
Cell growth and differentiation	neuronal regeneration related protein	NM_178096	*Nrep*	-1.11 (0.55)	0.023	-2.46	0.50 (0.49)	1.41	-1.46^*b*^ (1.36)	-2.74
Cell growth and differentiation	HOP homeobox	NM_133621	*Hopx*	-1.14 (0.52)	0.020	-2.20	0.60 (0.49)	1.51	-0.28 (0.71)	-1.21
Cell growth and differentiation	fibroblast growth factor 18	NM_019199	*Fgf18*	-1.12 (0.53)	0.021	-2.17	0.80 (0.57)	1.74	-0.51 (0.23)	-1.43
Cell growth and differentiation	ret proto-oncogene	NM_012643	*Ret*	-1.04 (0.14)	0.003	-2.06	0.63 (0.39)	1.55	-0.63 (0.15)	-1.55
Cell growth and differentiation	G0/G1 switch 2	NM_001009632	*G0s2*	-0.86 (0.21)	0.007	-1.82	0.58 (0.47)	1.50	0.26 (0.91)	1.20
Receptors and ion channels	adrenoceptor alpha 1D	NM_024483	*Adra1d*	-1.21 (0.52)	0.016	-2.31	1.12 (0.40)	2.17	-1.03^*b*^ (1.36)	-2.04
Receptors and ion channels	FXYD domain-containing ion transport regulator 3	NM_172317	*Fxyd3*	-1.01 (0.49)	0.023	-2.01	1.97 (0.48)	3.92	-0.36 (0.85)	-1.29
Receptors and ion channels	sodium channel, voltage-gated, type IV, beta	NM_001008880	*Scn4b*	-0.94 (0.30)	0.011	-1.92	0.38 (0.15)	1.30	-0.43 (0.49)	-1.35
Receptors and ion channels	transferrin receptor	NM_022712	*Tfrc*	-0.87 (0.21)	0.007	-1.83	0.09 (0.20)	1.06	-0.55 (0.32)	-1.46
Structural protein, cell adhesion	myosin binding protein C, fast-type	NM_001106257	*Mybpc2*	-0.93 (0.47)	0.026	-1.90	0.33 (0.52)	1.25	0.48 (0.82)	1.40
Structural protein, cell adhesion	protocadherin 17	NM_001107279	*Pcdh17*	-0.94 (0.44)	0.023	-1.91	0.90 (0.43)	1.86	-0.65 (0.43)	-1.57
Hormones	inhibin alpha	NM_012590	*Inha*	-0.89 (0.39)	0.020	-1.85	1.59 (0.79)	3.01	0.66 (0.21)	1.58
Transport	globin, alpha	NM_001013853	*LOC287167*	-1.64 (0.72)	0.015	-3.12	-0.23 (0.81)	-1.18	-1.80 (0.76)	-3.47
Transport	haemoglobin, beta adult major chain	NM_198776	*Hbb-b1*	-1.94 (0.75)	0.011	-3.83	-0.40 (0.77)	-1.32	-2.23 (0.76)	-4.71
Transport	alpha-2u globulin PGCL5	NM_147213	*LOC259245*	-0.87 (0.54)	0.047	-1.83	-0.03 (0.48)	-1.02	-0.12 (0.58)	-1.08
Others	uncharacterized LOC100909684	XR_146107	*LOC10099684*	-3.07 (1.43)	0.016	-8.43	2.55 (1.40)	5.85	-0.42 (0.76)	-1.34
Others	uncharacterized LOC100910110	XR_146304	*LOC100910110*	-1.94 (0.32)	0.003	-3.85	2.32 (0.31)	5.01	-0.46 (1.57)	-1.38

Values show gene expression. Log_2_ ratio reaching at least ±0.85 and *p* < 0.05 were considered as significant alterations

^a^RGD: rat genome database

^b^non significant change (*p* > 0.05)

**Table 3:** Genes significantly up-regulated in diabetes vehicle-treated vs. control vehicle-treated group on DNA microarray

				Diabetes Vehicle			Diabetes MVT		Control MVT	
				vs.			vs.		vs.	
				Control Vehicle			Diabetes Vehicle		Control Vehicle	
Gene function	Gene Name *provided by RGD* ^*a*^	Acc. Number	*Gene Symbol*	Log_2_ (SD)	Corr. *p*	Fold change	Log_2_ (SD)	Fold Change	Log_2_ (SD)	Fold Change
Metabolism	UDP-N-acetyl-alpha-D-galactosamine:polypeptide N-acetylgalactosaminyltransferase 15	XM_003752864	*Galnt15*	0.85 (0.53)	0.047	1.81	−0.58 (0.40)	−1.49	0.32 (0.55)	1.25
Metabolism	microtubule associated monooxygenase, calponin and LIM domain containing 1	NM_001106397	*Mical1*	0.89 (0.23)	0.007	1.86	−0.21 (0.19)	−1.16	0.52 (0.23)	1.43
Metabolism	proline dehydrogenase (oxidase) 1	NM_001135778	*Prodh*	0.95 (0.48)	0.026	1.93	−0.27 (0.63)	−1.21	0.93 (0.50)	1.91
Metabolism	4-hydroxyphenylpyruvate dioxygenase	NM_017233	*Hpd*	1.07 (0.38)	0.011	2.09	−0.06 (0.34)	−1.04	1.00 (0.48)	2.01
Stress response	glutathione S-transferase, theta 3	NM_001137643	*Gstt3*	0.88 (0.52)	0.042	1.84	−0.13 (0.38)	−1.09	0.61 (0.80)	1.52
Stress response	heme oxygenase (decycling) 1	NM_012580	*Hmox1*	1.08 (0.47)	0.017	2.12	−0.62 (0.49)	−1.54	0.52 (0.25)	1.43
Stress response	metallothionein 1a	NM_138826	*Mt1a*	1.32 (0.34)	0.005	2.49	−1.18 (0.43)	−2.26	0.09 (0.39)	1.06
Stress response	metallothionein 2a	NM_001137564	*Mt2a*	1.61 (0.34)	0.003	3.06	−1.70 (0.66)	−3.24	0.24 (0.53)	1.18
Immune response	NLR family member X1	NM_001025010	*Nlrx1*	0.87 (0.48)	0.036	1.83	−1.07 (0.43)	−2.10	0.30 (0.43)	1.23
Immune response	FK506 binding protein 5	NM_001012174	*Fkbp5*	1.13 (0.52)	0.020	2.19	−1.09 (0.32)	−2.13	0.83 (0.74)	1.77
Immune response	influenza virus NS1A binding protein	NM_001047085	*Ivns1abp*	1.27 (0.40)	0.008	2.41	−0.65 (0.35)	−1.57	0.68 (0.79)	1.60
Apoptosis/necrosis and inflammation	interleukin 6 receptor	NM_017020	*Il6r*	0.91 (0.50)	0.034	1.88	−1.00 (0.47)	−2.00	0.63 (0.67)	1.54
Cell growth and differentiation	H19, imprinted maternally expressed transcript	NR_027324	*H19*	0.92 (0.49)	0.032	1.89	−0.53 (0.47)	−1.44	1.16 (0.44)	2.24
Cell growth and differentiation	secreted frizzled-related protein 2	NM_001100700	*Sfrp2*	1.22 (0.73)	0.039	2.34	−0.62 (0.77)	−1.54	0.87^*b*^ (0.73)	1.83
Cell growth and differentiation	wingless-type MMTV integration site family, member 2B	NM_001191848	*Wnt2b*	1.32 (0.11)	0.003	2.50	−1.66 (0.48)	−3.15	0.16 (0.11)	1.11
Cell growth and differentiation	brain expressed, X-linked 1	NM_001037365	*Bex1*	1.41 (0.30)	0.003	2.65	−1.97 (0.45)	−3.92	0.25 (0.81)	1.19
Cell growth and differentiation	N-myc downstream regulated 1	NM_001011991	*Ndrg1*	0.85 (0.42)	0.027	1.81	−1.05 (0.33)	−2.07	0.41 (0.62)	1.33
Receptors and ion channels	solute carrier family 26 (anion exchanger), member 3	NM_053755	*Slc26a3*	0.88 (0.34)	0.015	1.84	−0.03 (0.23)	−1.02	0.01 (0.32)	1.01
Receptors and ion channels	ATPase, H^+^ transporting, lysosomal V1 subunit G2	NM_212490	*Atp6v1g2*	0.92 (0.41)	0.020	1.89	−1.05 (0.43)	−2.06	−0.75 (0.22)	−1.68
Receptors and ion channels	ATP-binding cassette, subfamily A (ABC1), member 1	NM_178095	*Abca1*	1.01 (0.35)	0.011	2.01	−0.22 (0.39)	−1.17	0.54 (0.40)	1.45
Receptors and ion channels	sarcolipin	NM_001013247	*Sln*	1.07 (0.61)	0.036	2.10	−0.23 (0.42)	−1.17	1.09 (0.61)	2.13
Signal transduction, regulation of transcription	zinc finger and BTB domain containing 16	NM_001013181	*Zbtb16*	0.89 (0.29)	0.011	1.85	−0.69 (0.33)	−1.62	0.37 (0.52)	1.30
Signal transduction, regulation of transcription	Rho-related BTB domain containing 1	NM_001107622	*Rhobtb1*	0.91 (0.25)	0.008	1.88	−0.22 (0.25)	−1.16	0.58 (0.55)	1.49
Signal transduction, regulation of transcription	connector enhancer of kinase suppressor of Ras 1	NM_001039011	*Cnksr1*	1.31 (0.67)	0.025	2.48	−1.64 (0.38)	−3.13	0.39 (0.72)	1.31
Signal transduction, regulation of transcription	cytochrome P450, family 26, subfamily b, polypeptide 1	NM_181087	*Cyp26b1*	1.62 (0.28)	0.003	3.08	−1.68 (0.47)	−3.20	0.25 (0.30)	1.19
Structural protein, cell adhesion	pannexin 2	NM_199409	*Panx2*	0.97 (0.17)	0.003	1.96	−0.56 (0.33)	−1.47	0.81 (0.15)	1.75
Structural protein, cell adhesion	myosin light chain kinase 2	NM_057209	*Mylk2*	1.17 (0.32)	0.006	2.25	−0.05 (0.37)	−1.03	1.05^*b*^ (0.69)	2.08
Hormones	resistin	NM_144741	*Retn*	1.08 (0.60)	0.034	2.12	−1.03 (0.44)	−2.05	0.51 (0.96)	1.43
Hormones	galanin/GMAP prepropeptide	NM_033237	*Gal*	2.41 (1.15)	0.018	5.31	−2.99 (0.92)	−7.96	1.82^*b*^ (1.20)	3.53
Hormones	natriuretic peptide A	NM_012612	*Nppa*	1.23 (0.45)	0.011	2.34	−0.62 (0.42)	−1.54	0.79 (0.38)	1.73
Others	myosin binding protein H-like	NM_001014042	*Mybphl*	1.26 (0.75)	0.038	2.39	−0.66 (0.68)	−1.58	1.09^*b*^ (0.71)	2.13
Others	prostaglandin D2 synthase (brain)	NM_013015	*Ptgds*	1.32 (0.69)	0.026	2.50	−0.64 (0.46)	−1.56	0.67 (0.87)	1.59
Others	visinin-like 1	NM_012686	*Vsnl1*	1.49 (0.79)	0.026	2.80	−1.01^*b*^ (0.77)	−2.01	1.16^*b*^ (0.76)	2.24
Others	transmembrane protein 140	NM_001009709	*Tmem140*	1.49 (0.27)	0.003	2.80	−0.59 (0.42)	−1.50	0.30 (0.26)	1.23
Others	IQ motif and ubiquitin domain containing	NM_001034130	*Iqub*	0.94 (0.36)	0.014	1.91	0.23 (0.20)	42021	0.52 (0.59)	1.44
Others	similar to apolipoprotein L2; apolipoprotein L-II	NM_001134801	*RGD1309808*	4.33 (2.91)	0.049	42358	−6.07 (0.68)	−67.24	1.61^*b*^ (3.76)	3.05
Others	thioredoxin domain containing 16	XM_001072487	*Txndc16*	0.85 (0.31)	0.015	1.80	−0.72 (0.32)	−1.65	0.51 (0.32)	1.42

Values show gene expression. Log_2_ ratio reaching at least ±0.85 and *p* < 0.05 were considered as significant alterations

^a^RGD: rat genome database

^b^non significant change (*p* > 0.05)

### Identification of genes associated with MVT-treatment in DM

**Fig. 2:**
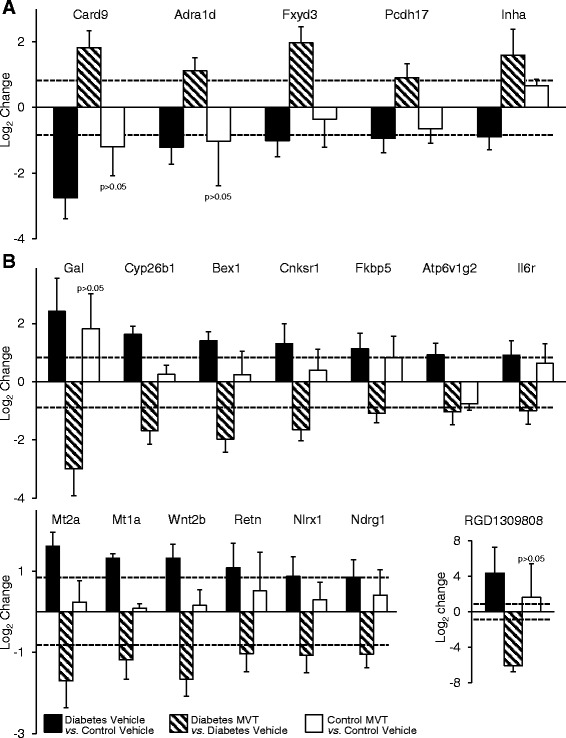
*Genes with significantly and oppositely altered expression in diabetes vehicle-treated* vs. *diabetes MVT-treated group.* **a** Significantly *down-*regulated genes in diabetes vehicle-treated group vs. control vehicle-treated group which are *up-*regulated in diabetes MVT-treated group vs. diabetes vehicle treated-group. No significant change in control MVT-treated group vs. control vehicle-treated group. **b** Significantly *up-*regulated genes in diabetes vehicle-treated group vs. control vehicle-treated group which are *down-*regulated in diabetes MVT-treated group vs. diabetes vehicle-treated group. No significant change in control MVT-treated group vs. control vehicle-treated group. Values are expressed as mean ± S.E.M. Log_2_ ratio reaching at least ±0.85 (represented with dotted lines) and *p* < 0.05 were considered as significant alterations

To determine genes associated with the effects of MVT-treatment in diabetic hearts, cardiac gene expression induced in the diabetic MVT-treated group was compared with the diabetic vehicle-treated group (Tables 4 and 5). In diabetic MVT-treated hearts, 15 genes showed significant up-regulation and 29 genes showed significant down-regulation, compared to diabetic vehicle-treated controls (Tables 4 and 5). In the diabetic MVT-treated group, an additional 23 genes were found, which expression pattern was significantly influenced only by the MVT-treatment. These 23 genes did not show significant gene expression change in the diabetic vehicle-treated group as compared to the control vehicle-treated group (Tables 2 and 5). To assess potential beneficial effects of MVT-treatment, we analysed opposite gene expression changes in the diabetic MVT-treated group as compared to the diabetic vehicle-treated group. Among the oppositely altered genes in the diabetic MVT-treated group, 5 genes showed significant up-regulation and 14 genes showed significant down-regulation, respectively (Fig. 2). These 19 genes may be associated with potential cardioprotective effects of MVT-treatment in DM.

**Table 4:** Genes significantly down-regulated in diabetes MVT-treated vs. diabetes vehicle-treated group on DNA microarray

				Diabetes MVT			Diabetes Vehicle		Control MVT	
				vs.			vs.		vs.	
				Diabetes Vehicle			Control Vehicle		Control Vehicle	
Gene function	Gene Name *provided by RGD* ^*a*^	Acc. Number	*Gene Symbol*	Log_2_ (SD)	Corr. *p*	Fold change	Log_2_ (SD)	Fold Change	Log_2_ (SD)	Fold Change
Metabolism	dipeptidase 1 (renal)	L07315	*Dpep1*	−1.02 (0.27)	0.007	−2.03	0.79 (0.47)	1.72	−0.17 (0.50)	−1.12
Metabolism	glycosylphosphatidylinositol anchored high density lipoprotein binding protein 1	NM_001130547	*Gpihbp1*	−0.89 (0.18)	0.006	−1.86	0.59 (0.33)	1.50	0.10 (0.37)	1.07
Metabolism	flavin containing monooxygenase 2	NM_144737	*Fmo2*	−0.85 (0.36)	0.028	−1.80	0.80 (0.24)	1.74	0.12 (0.29)	1.08
Stress response	metallothionein 2a	NM_001137564	*Mt2a*	−1.70 (0.66)	0.018	−3.24	1.61 (0.34)	3.06	0.24 (0.53)	1.18
Stress response	metallothionein 1a	NM_138826	*Mt1a*	−1.18 (0.43)	0.017	−2.26	1.32 (0.34)	2.49	0.09 (0.39)	1.06
Immune response	FK506 binding protein 5	NM_001012174	*Fkbp5*	−1.09 (0.32)	0.008	−2.13	1.13 (0.52)	2.19	0.83 (0.74)	1.77
Immune response	NLR family member X1	NM_001025010	*Nlrx1*	−1.07 (0.43)	0.023	−2.10	0.87 (0.48)	1.83	0.30 (0.43)	1.23
Apoptosis/necrosis and inflammation	chemokine (C-X-C motif) ligand 13	NM_001017496	*Cxcl13*	−1.66 (0.84)	0.036	−3.17	0.61 (1.02)	1.53	0.18 (0.96)	1.14
Apoptosis/necrosis and inflammation	interleukin 6 receptor	NM_017020	*Il6r*	−1.00 (0.47)	0.033	−2.00	0.91 (0.50)	1.88	0.63 (0.67)	1.54
Cell growth and differentiation	brain expressed, X-linked 1	NM_001037365	*Bex1*	−1.97 (0.45)	0.004	−3.92	1.41 (0.30)	2.65	0.25 (0.81)	1.19
Cell growth and differentiation	wingless-type MMTV integrationsite family, member 2B	NM_001191848	*Wnt2b*	−1.66 (0.48)	0.007	−3.15	1.32 (0.11)	2.50	0.16 (0.11)	1.11
Cell growth and differentiation	RAB3 GTPase activating protein subunit 2	AI072072	*Rab3gap2*	−1.25 (0.57)	0.029	−2.38	0.53 (0.33)	1.45	0.22 (0.53)	1.17
Cell growth and differentiation	N-myc downstream regulated 1	NM_001011991	*Ndrg1*	−1.05 (0.33)	0.012	−2.07	0.85 (0.42)	1.81	0.41 (0.62)	1.33
Cell growth and differentiation	WNT1 inducible signaling pathway protein 2	NM_031590	*Wisp2*	−1.02 (0.53)	0.043	−2.02	0.60 (0.40)	1.52	0.47 (0.34)	1.39
Cell growth and differentiation	serine (or cysteine) peptidase inhibitor, clade A, member 3 N	NM_031531	*Serpina3n*	−0.90 (0.48)	0.048	−1.87	−0.03 (0.39)	−1.02	0.90 (1.09)	1.87
Receptors and ion channels	ATPase, H^+^ transporting,lysosomal V1 subunit G2	NM_212490	*Atp6v1g2*	−1.05 (0.43)	0.024	−2.06	0.92 (0.41)	1.89	−0.75 (0.22)	−1.68
Receptors and ion channels	melanocortin 2 receptoraccessory protein	NM_001135834	*Mrap*	−0.92 (0.42)	0.033	−1.89	0.65 (0.19)	1.56	0.12 (0.21)	1.09
Signal transduction, regulation of transcription	cytochrome P450, family 26, subfamily b, polypeptide 1	NM_181087	*Cyp26b1*	−1.68 (0.47)	0.007	−3.20	1.62 (0.28)	3.08	0.25 (0.30)	1.19
Signal transduction, regulation of transcription	connector enhancer of kinase suppressor of Ras 1	NM_001039011	*Cnksr1*	−1.64 (0.38)	0.004	−3.13	1.31 (0.67)	2.48	0.39 (0.72)	1.31
Structural protein, cell adhesion	solute carrier family 43 (amino acid system L transporter), member 2	NM_001105812	*Slc43a2*	−1.06 (0.39)	0.019	−2.09	0.34 (0.19)	1.27	−0.07 (0.18)	−1.05
Hormones	galanin/GMAP prepropeptide	NM_033237	*Gal*	−2.99 (0.92)	0.007	−7.96	2.41 (1.15)	5.31	1.82 (1.20)	3.53
Hormones	resistin	NM_144741	*Retn*	−1.03 (0.44)	0.025	−2.05	1.08 (0.60)	2.12	0.51 (0.96)	1.43
Others	similar to apolipoprotein L2; apolipoprotein L-II	NM_001134801	*RGD1309808*	−6.07 (0.68)	<0.001	−67.24	4.33 (2.91)	20.12	1.61 (3.76)	3.05
Others	secreted phosphoprotein 1	NM_012881	*Spp1*	−1.35 (0.59)	0.025	−2.55	−0.40 (0.77)	−1.32	2.30 (1.49)	4.93
Others	elongation of very long chain fatty acids protein 6-like	XM_003749393	*LOC100910695*	−1.01 (0.39)	0.021	−2.02	0.33 (0.43)	1.25	−0.94 (0.55)	−1.92
Others	WW domain binding protein 5	NM_001127502	*Wbp5*	−0.99 (0.20)	0.005	−1.99	0.54 (0.15)	1.45	0.07 (0.38)	1.05
Others	coiled-coil domain containing 136	XM_001064000	*Ccdc136*	−0.98 (0.10)	0.002	−1.98	0.62 (0.18)	1.53	0.09 (0.39)	1.06
Others	multimerin 1	XM_001071128	*Mmrn1*	−0.91 (0.38)	0.027	−1.88	−0.31 (0.36)	−1.24	−0.14 (0.31)	−1.10
Others	epsin 3	NM_001024791	*Epn3*	−0.87 (0.44)	0.043	−1.82	0.46 (0.50)	1.37	−0.16 (0.32)	−1.12

Values show gene expression. Log_2_ ratio reaching at least ±0.85 and *p* < 0.05 were considered as significant alterations

^a^RGD: rat genome database

^b^non significant change (*p* > 0.05)

**Table 5:** Genes significantly up-regulated in diabetes MVT-treated vs. diabetes vehicle-treated group on DNA microarray

				Diabetes MVT			Diabetes Vehicle		Control MVT	
				vs.			vs.		vs.	
				Diabetes Vehicle			Control Vehicle		Control Vehicle	
Gene function	Gene Name *provided by RGD* ^*a*^	Acc. Number	*Gene Symbol*	Log_2_ (SD)	Corr. *p*	Fold change	Log_2_ (SD)	Fold Change	Log_2_ (SD)	Fold Change
Metabolism	L-2-hydroxyglutarate dehydrogenase	NM_001108028	*L2hgdh*	1.06 (0.49)	0.032	2.09	−0.52 (1.01)	−1.44	0.04 (0.92)	1.03
Apoptosis/necrosis and inflammation	chemokine (C-X-C motif) ligand 9	NM_145672	*Cxcl9*	1.03 (0.40)	0.021	2.04	−0.29 (0.60)	−1.22	−0.19 (0.81)	−1.14
Apoptosis/necrosis and inflammation	caspase recruitment domain family, member 9	NM_022303	*Card9*	1.82 (0.52)	0.007	3.53	−2.74 (0.64)	−6.69	−1.20^*b*^ (0.88)	−2.29
Receptors and ion channels	adrenoceptor alpha 1D	NM_024483	*Adra1d*	1.12 (0.40)	0.017	2.17	−1.21 (0.52)	−2.31	−1.03^*b*^ (1.36)	−2.04
Receptors and ion chanells	FXYD domain-containing ion transport regulator 3	NM_172317	*Fxyd3*	1.97 (0.48)	0.004	3.92	−1.01 (0.49)	−2.01	−0.36 (0.85)	−1.29
Signal transduction, regulation of transcription	regulator of telomere elongation helicase 1	NM_001191857	*Rtel1*	1.14 (0.27)	0.006	2.20	−0.71 (1.02)	−1.64	0.58 (1.14)	1.49
Signal transduction, regulation of transcription	staufen double-stranded RNA binding protein 2	NM_001007149	*Stau2*	1.36 (0.71)	0.042	2.56	−0.38 (0.27)	−1.30	0.22 (0.41)	1.16
Structural protein, cell adhesion	protocadherin 17	NM_001107279	*Pcdh17*	0.90 (0.43)	0.036	1.86	−0.94 (0.44)	−1.91	−0.65 (0.43)	−1.57
Hormones	inhibin alpha	NM_012590	*Inha*	1.59 (0.79)	0.035	3.01	−0.89 (0.39)	−1.85	0.66 (0.21)	1.58
Others	similar to HTGN29 protein; keratinocytes associated transmembrane protein 2	NM_001106999	*RGD131352*	1.05 (0.48)	0.032	2.07	0.29 (0.49)	1.23	0.49 (0.53)	1.41
Others	WDNM1 homolog	NM_001003706	*LOC360228*	1.32 (0.63)	0.033	2.50	−0.59 (0.77)	−1.50	1.01^*b*^ (0.67)	2.01
Others	ring finger protein 135	NM_001012010	*Rnf135*	0.86 (0.33)	0.023	1.82	−0.44 (0.08)	−1.35	−0.06 (0.46)	−1.05
Others	uncharacterized LOC100910110	XR_146304	*LOC100910110*	2.32 (0.31)	0.001	5.01	−1.94 (0.32)	−3.85	−0.46 (1.57)	−1.38
Others	uncharacterized LOC100909684	XR_146107	*LOC10099684*	2.55 (1.40)	0.045	5.85	−3.07 (1.43)	−8.43	−0.42 (0.76)	−1.34
Others	serine hydrolase-like 2	NM_001130579	*Serhl2*	5.17 (0.48)	<0.001	35.96	−3.62^*b*^ (2.81)	−12.31	−3.45^*b*^ (2.82)	−10.92

Values show gene expression. Log_2_ ratio reaching at least ±0.85 and *p* < 0.05 were considered as significant alterations

^a^RGD: rat genome database

^b^non significant change (*p* > 0.05)

### Identification of genes associated with MVT-treatment in healthy condition

To determine genes associated with the effects of MVT-treatment in healthy control hearts, cardiac gene expression induced in the control MVT-treated group was compared with the control vehicle-treated group (Tables 6 and 7). In control MVT-treated hearts, 18 genes showed significant up-regulation and 6 genes showed significant down-regulation (Tables 6 and 7). Out of these significantly altered 24 genes, 18 genes were not significantly altered in diabetic MVT-treated rats. These 18 genes may be associated with potential beneficial effects of MVT-treatment in healthy or diseased conditions.

**Table 6:** Genes significantly down-regulated in control MVT-treated vs. control vehicle-treated group on DNA microarray

				Control MVT			Diabetes Vehicle		Diabetes MVT	
				vs.			vs.		vs.	
				Control Vehicle			Control Vehicle		Diabetes Vehicle	
Gene function	Gene Name *provided by RGD* ^*a*^	Acc. Number	*Gene Symbol*	Log_2_ (SD)	Corr. *p*	Fold change	Log_2_ (SD)	Fold Change	Log_2_ (SD)	Fold Change
Stress response	APEX (apurinic/apyrimidinic) endonuclease) 2	NM_001079892	*Apex2*	−1.18 (0.63)	0.043	−2.26	−0.94 (0.56)	−1.92	0.09 (0.26)	1.06
Receptors and ion channels	sodium leak channel, non-selective	NM_153630	*Nalcn*	−0.85 (0.44)	0.043	−1.80	−0.59 (0.36)	−1.50	0.16 (0.41)	1.12
Signal transduction, regulation of transcription	triggering receptor expressed on myeloid cells 2	NM_001106884	*Trem2*	−1.03 (0.40)	0.021	−2.05	−0.21 (0.88)	−1.15	−0.98 (0.94)	−1.98
Transport	hemoglobin, beta adult major chain	NM_198776	*Hbb-b1*	−2.23 (0.76)	0.016	−4.71	−1.94 (0.75)	−3.83	−0.40 (0.77)	−1.32
Transport	globin, alpha	NM_001013853	*LOC287167*	−1.80 (0.76)	0.020	−3.47	−1.64 (0.72)	−3.12	−0.23 (0.81)	−1.18
Others	EF-hand calcium binding domain 6	XM_001077962	*Efcab6*	−1.66 (0.62)	0.017	−3.15	0.40 (0.99)	1.32	−1.36^*b*^ (1.38)	−2.57

Values show gene expression. Log_2_ ratio reaching at least ±0.85 and *p* < 0.05 were considered as significant alterations

^a^RGD: rat genome database

^b^non significant change (*p* > 0.05)

**Table 7:** Genes significantly up-regulated in control MVT-treated vs. control vehicle-treated group on DNA microarray

				Control MVT			Diabetes Vehicle		Diabetes MVT	
				vs.			vs.		vs.	
				Control Vehicle			Control Vehicle		Diabetes Vehicle	
Gene function	Gene Name *provided by RGD* ^*a*^	Acc. Number	*Gene Symbol*	Log_2_ (SD)	Corr. *p*	Fold change	Log_2_ (SD)	Fold Change	Log_2_ (SD)	Fold Change
Metabolism	proline dehydrogenase (oxidase) 1	NM_001135778	*Prodh*	0.93 (0.50)	0.043	1.91	0.95 (0.48)	1.93	−0.27 (0.63)	−1.21
Metabolism	enolase 2, gamma, neuronal	NM_139325	*Eno2*	1.00 (0.30)	0.017	2.00	0.98^*b*^ (0.85)	1.97	−1.32^*b*^ (1.12)	−2.50
Metabolism	4-hydroxyphenylpyruvate dioxygenase	NM_017233	*Hpd*	1.00 (0.48)	0.034	2.01	1.07 (0.38)	2.09	−0.06 (0.34)	−1.04
Immune response	complement factor B	NM_212466	*Cfb*	0.85 (0.32)	0.025	1.80	0.14 (0.34)	1.10	0.22 (0.34)	1.17
Immune response	2’-5’ oligoadenylate synthetase 1A	NM_138913	*Oas1a*	0.92 (0.33)	0.020	1.90	0.40 (0.28)	1.32	−0.43 (0.24)	−1.35
Immune response	2-5 oligoadenylate synthetase 1B	AF068268	*Oas1b*	1.04 (0.18)	0.015	2.06	−0.52 (0.50)	−1.44	0.11 (0.83)	1.08
Immune response	2’-5’ oligoadenylate synthetase 1I	NM_001009680	*Oas1i*	0.87 (0.24)	0.018	1.83	0.13 (0.31)	1.09	−0.29 (0.22)	−1.22
Immune response	interferon regulatory factor 7	NM_001033691	*Irf7*	1.13 (0.27)	0.016	2.18	−0.05 (0.12)	−1.04	0.25 (0.20)	1.19
Immune response	complement component 4A (Rodgers blood group)	NM_031504	*C4a*	1.18 (0.39)	0.017	2.27	0.29 (0.44)	2.27	0.05 (0.34)	1.04
Immune response	hepcidin antimicrobial peptide	NM_053469	*Hamp*	1.38 (0.74)	0.040	2.60	0.59 (0.77)	1.51	−0.56 (0.74)	−1.47
Immune response	myxovirus (influenza virus) resistance 2	NM_134350	*Mx2*	2.14 (0.95)	0.020	4.41	0.42 (0.63)	1.34	−0.72 (0.67)	−1.65
Cell growth and differentiation	neuronatin	NM_053601	*Nnat*	0.99 (0.41)	0.025	1.98	0.73 (0.40)	1.66	−0.06 (0.74)	−1.04
Cell growth and differentiation	H19, imprinted maternally expressed transcript	NR_027324	*H19*	1.16 (0.44)	0.020	2.24	0.92 (0.49)	1.89	−0.53 (0.47)	−1.44
Receptors and ion channels	ATPase, H^+^/K^+^ exchanging, alpha polypeptide	NM_012509	*Atp4a*	0.86 (0.19)	0.017	1.81	0.45 (0.48)	1.37	−0.05 (0.59)	−1.04
Receptors and ion channels	sarcolipin	NM_001013247	*Sln*	1.09 (0.61)	0.043	2.13	1.07 (0.61)	2.10	−0.23 (0.42)	−1.17
Receptors and ion channels	ATPase, Na^+^/K^+^ transporting, beta 4 polypeptide	NM_053381	*Atp1b4*	1.14 (0.63)	0.043	2.21	0.41 (0.64)	1.33	0.04 (0.81)	1.03
Others	2’-5’ oligoadenylate synthetase-like 2	NM_001009682	*Oasl2*	0.96 (0.34)	0.020	1.95	−0.21 (0.19)	−1.15	0.08 (0.14)	1.05
Others	GRAM domain containing 3	NM_001014011	*Gramd3*	0.97 (0.31)	0.018	1.96	−0.03 (0.41)	−1.02	0.36 (0.79)	1.29

Values show gene expression. Log_2_ ratio reaching at least ±0.85 and *p* < 0.05 were considered as significant alterations

^a^RGD: rat genome database

^b^non significant change (*p* > 0.05)

### Validation of microarray data by qRT-PCR

To confirm the microarray data we measured the expression of selected 5 genes by qRT-PCR in 2 different setups: 1) diabetes vehicle-treatment vs. control vehicle-treatment groups and 2) diabetes MVT-treatment vs. diabetes vehicle-treatment groups (Tables 8 and 9). In both setups, the expression changes of 4 genes were confirmed by qRT-PCR and showed reliability of the microarray data.

**Table 8:** Primers to qRT-PCR

Gene name	Gene symbol	Acc. Number	Forward	Length	Reverse	Length
metallothionein 1a	Mt1a	NM_138826	caccagatctcggaatggac	20	aggagcagcagctcttcttg	20
metallothionein 2a	Mt2a	NM_001137564	catggaccccaactgctc	18	aggtgcatttgcattgtttg	20
cytochrome P450, family 26, subfamily b, polypeptide 1	Cyp26b1	NM_181087	acggcaaggagatgacca	18	gcataggctgcgaagatca	19
galanin prepropeptide	Gal	NM_033237	tggagtttctcagtttcttgcac	23	ggtgtggtctcaggactgct	20
connector enhancer of kinase suppressor of Ras 1	Cnksr1	NM_001039011	tgtggctgggatctgtcac	19	tgctggtggtgtgaatttct	20

**Table 9:** qRT-PCR results

			Diabetes Vehicle vs. Control Vehicle					
Description	Gene symbol	Acc. Number	DNA MICROARRAY		qRT-PCR			confirmed
			fold change	p value	log_2_ ratio (SD)	fold change	regulation	
metallothionein 1a	Mt1a	NM_138826	2.49	0.005	2.36 (1.35)	5.13	up	*yes*
metallothionein 2a	Mt2a	NM_001137564	3.06	0.003	2.36 (0.93)	5.12	up	*yes*
cytochrome P450, family 26, subfamily b, polypeptide 1	Cyp26b2	NM_181087	3.08	0.003	2.50 (0.85)	5.65	up	*yes*
galanin/GMAP prepropeptide	Gal	NM_033237	5.31	0.018	2.69 (0.86)	6.43	up	*yes*
connector enhancer of kinase suppressor of Ras 1	Cnksr2	NM_001039011	2.48	0.025	0.002 (0.26)	1.00	no change	no
			Diabetes MVT vs. Diabetes Vehicle					
Description	Gene symbol	Acc. Number	DNA MICROARRAY		qRT-PCR			confirmed
			fold change	p value	log_2_ ratio (SD)	fold change	regulation	
metallothionein 1a	Mt1a	NM_138826	−2.26	0.017	−1,44 (0.91)	−2.71	down	*yes*
metallothionein 2a	Mt2a	NM_001137564	−3.24	0.018	−2.53 (0.68)	−5.76	down	*yes*
cytochrome P450, family 26, subfamily b, polypeptide 1	Cyp26b2	NM_181087	−3.20	0.007	−2.91 (0.32)	−7.50	down	*yes*
galanin/GMAP prepropeptide	Gal	NM_033237	−7.96	0.007	−2.72 (0.64)	−6.60	down	*yes*
connector enhancer of kinase suppressor of Ras 1	Cnksr2	NM_001039011	−3.13	0.004	−0.11 (0.33)	1.08	no change	no

MVT = preparation of minerals, vitamins, and trace elements

## Discussion

In the present study we have confirmed that chronic treatment with a MVT preparation attenuated the progression of DM by improving diagnostic markers of DM including glucose tolerance, FBG, HbA1c, and serum insulin levels in male diabetic rats [14]. We have shown here that the development of DM induces marked alterations in the cardiac gene expression pattern. This is the first demonstration that a MVT preparation significantly altered the myocardial [38, 39] gene expression pattern by altering transcript levels of several genes in both diabetic and healthy rats. The significantly altered genes can be classified into different clusters (e.g. metabolism; stress response; immune response; cell growth and differentiation; ion channels and receptors; signal transduction and regulation of transcription; structural proteins and cell adhesion; hormones; transport; etc.). Some of these genes are related to the development of diabetic cardiomyopathy (e.g. cardiac hypertrophy and fibrosis, stress response, hormones associated with insulin resistance, etc.). Moreover, some other genes without any definite function in the myocardium were also changed in response to DM or MVT-treatment.

### Genes associated with diabetic cardiomyopathy

One of the major cardiovascular complications of DM is diabetic cardiomyopathy (DCM) [40, 41], which is defined as left ventricular dysfunction with hypertrophy and fibrosis in the absence of hypertension, coronary artery disease and valvular or congenital heart disease [42]. The complex underlying molecular mechanisms of the above-mentioned functional and morphologic changes have been intensively investigated [40–42]. In our present study, we have shown altered expression of several genes related to cardiac hypertrophy and remodelling in accordance with the literature (e.g. down-regulation of *caspase recruitment domain family*, *member 9 (Card9)* [43] and *adrenoceptor alpha 1d (adra1d)* [44]; up-regulation of the angiogenesis inductor *cytochrome P450, family 26, subfamily B, polypeptide 1 (Cyp26b1)* [45, 46]; *FXYD domain containing ion transport regulator 3 (Fxyd3)* also called phospholemman-like protein potentially regulating Na^+^/K^+^/ATP-ase activity [47–49]; *ATPase, H*^*+*^*Transporting, Lysosomal 13 kDa, V1 Subunit G21 (Atp6v1g2)* [50] related to hepatitis C virus-associated dilated cardiomyopathy; and a well-known marker of hypertrophy and heart failure, *natriuretic peptide A* (Nppa) [40, 41] (Tables 2 and 3). In our present study, another group of genes altered in response to DM is involved in cell proliferation in different organs (e.g. down-regulation of the antiproliferative and tumour suppressor *protocadherin-17* (Pcdh17) [51] and up-regulation of the insulin signalling pathway promoter *connector enhancer of kinase suppressor of Ras 1 (Cnksr1)* [52, 53]; *and wingless-type MMTV integration site family, member 2B (Wnt2b)* playing a role in pancreatic beta cell replication [54] (Tables 2 and 3)*.* These aforementioned studies, in agreement with our present study, suggest that altered metabolic parameters in DM may induce cardiac gene expression changes leading to the induction of general mechanisms of cell proliferation and cardiac hypertrophy. Nevertheless, these aforementioned genes have not previously been shown to play a role in the development of diabetic cardiomyopathy. In our present study, MVT-treatment in diabetic rats resulted in opposite gene expression changes in case of some aforementioned genes (e.g. *caspase recruitment domain family, member 9 (Card9); adrenoceptor alpha 1d (adra1d); cytochrome P450, family 26, subfamily B, polypeptide 1 (Cyp26b1; FXYD domain containing ion transport regulator 3 (Fxyd3); ATPase, H*^*+*^*Transporting, Lysosomal 13 kDa, V1 Subunit G21 (Atp6v1g2);* etc.) showing a beneficial effect of MVT-treatment on the development of diabetic cardiomyopathy (Tables 2 and 5, Fig. 2). Although we have not characterized diabetic cardiomyopathy in our present study, STZ-treated rats are well known to develop this cardiovascular complication at ages similar to that of used in the present study [19].

### Genes associated with increased oxidative/nitrative stress in DM

Increased cardiovascular oxidative and nitrative stress is another well-known factor in the development of diabetic cardiomyopathy [40, 41]. In the present study, members of another functional gene cluster related to oxidative/nitrative stress and stress response showed altered expression in diabetic vehicle-treated hearts as compared to controls, in accordance with literature data (e.g. up-regulation of the zinc ion containing antioxidative *metallothionein 1a (Mt1a)* and *metallothionein 2a (Mt2a*) [55, 56]; the cardiovascular risk factor *interleukin-6 receptor (Ilr6)* [57, 58]; the antioxidative *heme oxygenase (decycling) 1 (Hmox1)* [59]; and *glutathione S-transferase, theta 3 (Gstt3)* [60]) (Tables 2 and 3). Glutathione S-transferase catalyzes the conjugation of reduced glutathione on a wide variety of substrates [60] including reactive oxygen and nitrogen species [61]. Interestingly, we have found here the overexpression of glutathione S-transferase in DM similar to the up-regulation of this gene in metabolic syndrome [23] and cholesterol diet-induced hyperlipidaemia [62] in our previous studies. Our results suggest that up-regulation of antioxidative genes including *glutathione S-transferase; heme oxygenase 1; metallothionein 1a* and *2a* may be an adaptive response in DM to antagonize elevated oxidative/nitrative stress in the myocardium. In contrast, decreased expression of metallothionein proteins have been reported in the heart [63] and the aorta [64] in experimental diabetes. Moreover, it has been published that the expression of metallothioneins could be increased by a chelator-regulated restoration of copper regulation [63] and zinc supplementation in experimental diabetes [64, 65]. Nevertheless, it should be noted that these aforementioned studies used different types of diabetes models (a genetic T1DM model OVE26 mice and single injection of STZ in adult mice or rats) with more severe hyperglycaemia than that developed in our model in the present study. In addition, we have previously shown marked differences in gene expression profiles in vascular and cardiac tissues in response to nitrate tolerance, which indicates different tissue-specific signalling pathways in different organs [66]. MVT-treatment in diabetic rats showed opposite gene expression changes in the cases of the aforementioned genes (Tables 2 and 5, Fig. 2). This could be explained by the beneficial effect of MVT-treatment on the severity of DM and potentially reduced oxidative/nitrative stress.

### Genes associated with insulin resistance

Insulin resistance is another well-known phenomenon in diabetic cardiomyopathy [40, 41]. Although the precise mechanisms by which cardiac insulin resistance developed in DM are poorly characterized, numerous metabolic and hormonal changes in the diabetic heart have been demonstrated [19–22, 40, 41]. In this present study, we have shown altered expression of several genes related to insulin resistance in the hearts of diabetic rats (e.g. up-regulation of *resistin (Retn)* responsible for induction of cardiac insulin resistance in rodents and chronic inflammation in humans [67] and *FK506 binding protein 5 (Fkbp5)* associated with decreased ligand sensitivity of the glucocorticoid receptor [68]. Interestingly, here we have shown up-regulation of *galanin/GMAP prepropeptide (Gal)* which has trophic effects on cells and increases insulin sensitivity in the diabetic heart [69]. Increased expression of galanin might be a counter-regulatory mechanism against insulin resistance in the diabetic heart. Surprisingly, a regulatory hormone of the reproductive function, *inhibin 1-alpha (Inha)* [70] showed significant up-regulation in the present study. Association of this gene with heart or DM has never been shown previously.

### Genes not associated with DM before

Some of the genes showing altered expression in diabetic hearts in the present study have not yet been related to diabetic cardiomyopathy (e.g. up-regulation of *prostaglandin b2 synthase (brain) (Ptgds); fibroblast growth factor (Fgf18)* and down-regulation *of HOP homeobox (Hopx); neuronal regeneration related protein (Nrep);* etc.*).* Interestingly, we have found here the overexpression of *brain expressed X-linked 1 (Bex1)* in DM similar to the up-regulation of this gene in metabolic syndrome [23] in our previous study. Some other altered genes were not classified into specific functional clusters or indicated as yet uncharacterized, predicted genes and fragments (e.g. up-regulation of *RGD1309808* also called *similar to apolipoprotein L2* or down-regulation of *uncharacterized LOC100909684* and *LOC100910110* genes*)*, the relevance of which should not be ignored.

### Genes altered in healthy condition due to MVT-treatment

Regular consumption of MVT preparations as medical food for diabetics is common in developed countries. However, preclinical or clinical evaluation of such preparations is surprisingly limited in the literature [12–16]. To the best of our knowledge, none of the investigated and significantly altered genes in this study has been reported to show altered expression, either in healthy or in diabetic hearts in response to MVT-treatment. In the present study, 24 genes showed significant alteration in control MVT-treated hearts as compared to control vehicle-treated hearts (Tables 5 and 6), however, we did not observe any phenotypic changes in this group. Out of the 24 aforementioned genes, 18 were not significantly altered in diabetic MVT-treated rats compared to the diabetes vehicle-treated ones; therefore, these genes might be associated to the beneficial effects of MVT-treatment observed in the diabetic group. Accordingly, a major cluster of significantly altered cardiac genes in response to MVT treatment in control animals was associated with immune and antimicrobial response (e.g. *complement factor B (Cfb); complement component 4a (C4a); interferon regulatory factor 7 (irf7)*; *hepcidin antimicrobial peptide (Hamp)*; *myxovirus (influenza virus) resistance 2 (Mx2)*; and viral RNA degradation regulators including *2′-5′′ oligoadenylate synthetase 1A; 1B; and 1 L;* as well as *2′-5′ oligoadenylate synthetase-like 2*) which is in line with the known immune system boosting effect of some of the components of the MVT preparation such as e.g. selenium [71].

### Limitations

Our study is not without limitations. Based on our present results one may not be able to differentiate entirely between the effects of diabetes and postnatal development due to insulin insufficiency, however, this model may also have clinical significance in DM in the pediatric age [72]. Furthermore, cardiac morphological and functional parameters to verify the development of diabetic cardiomyopathy were not investigated in this study; however, the neonatal STZ-injected rat is a well-characterized model of diabetes with cardiovascular complications including hypertension and LV hypertrophy with decreased cardiac function at a similar age to that used in our present study [28–34]. Although our study does not specify which cell type (i.e. cardiomyocyte, fibroblast, smooth muscle cell, etc.) may be responsible for the observed alterations of cardiac gene expression due to DM, the contribution of cardiomyocytes is likely the most significant [38, 39]. Although longer ex vivo heart perfusion was reported to alter cardiac gene expression profile [73], it is unlikely that 10 min perfusion in our present study significantly affected gene expression profile, however, it still cannot be completely excluded that the stability of transcripts might have been influenced differentially by the crystalloid buffer used during ex vivo heart perfusion.

Our results regarding altered cardiac gene expression due to DM are based on determinations of 41012 cardiac transcript levels. Examination of the role of corresponding proteins was out of the scope of the present study; however, mechanistic data would strengthen our results. In addition, it is unclear whether significantly altered gene expression changes are causes or consequences in the development of diabetic cardiomyopathy. Moreover, it also needs to be further investigated whether the MVT-treatment in DM associated with opposite cardiac gene expression changes is the cause of attenuation of the severity of DM or a consequence of DM. Therefore, focused future studies are necessary to perform in-depth functional assessment of selected genes and specifically aim to investigate the precise role of these genes in the cardiac effects of diabetes mellitus and/or MVT-treatment. The results of the present study do not provide evidence on the mechanism of the MVT preparation and the different contributions of the 26 individual components. We assume that the potential interactions of these components and their combined effects rather than the value of a single component could be responsible for the effects of the MVT preparation on cardiac gene expression changes and the severity of DM; however, the effects of each component on gene expression or DM were not investigated in the present study. Indeed, a recent report [74] showing a reduction of cancer risk in humans by a daily intake of multivitamins and minerals suggests that the combined effect of multivitamins is more important in the beneficial effect than any single component.

## Conclusions

In summary, we have found that 12 week-old STZ-treated rats developed DM characterized by hyperglycaemia and impaired glucose tolerance. We have shown that the severity of DM could be attenuated by a complex MVT preparation. We have demonstrated for the first time that MVT-treatment is associated with profound modifications of the cardiac transcriptome in both healthy and diabetic conditions. In addition, several of the genes showing altered expression in the hearts of diabetic rats have not been implicated in DM previously. We conclude that DM alters the gene expression pattern of the myocardium, which may be involved in the development of cardiac pathologies in the state of DM and these pathological processes may be attenuated by MVT-treatment. Based on our exploratory results, future preclinical and clinical studies should be carried out to investigate the precise role of specific genes in the development of cardiac consequences of DM and MVT-treatment to obtain deeper mechanistic insight.

## Additional file

Additional file 1:
**Ingredients of the standard rat chow.**
